# Experimental Study on the Pore Structure Evolution of Sandstone Under Brine Erosion

**DOI:** 10.3390/ma18153500

**Published:** 2025-07-25

**Authors:** Kang Peng, Tao Wu, Kun Luo, Song Luo, Jiaqi Zhou, Yuanmin Wang

**Affiliations:** 1 School of Resources and Safety Engineering, Central South University, Changsha 410083, China; 235511037@csu.edu.cn (T.W.); luokun@csu.edu.cn (K.L.); 2 School of Resources Environment and Safety Engineering, University of South China, Hengyang 421001, China; luosong@usc.edu.cn; 3 Changchun Gold Research Institute Co., Ltd., Changchun 130012, China; zhoujiaqi@chncgri.cn

**Keywords:** brine erosion, red sandstone, NMR test, pore structure

## Abstract

The mechanical properties of sandstone, a common building material, are influenced by a variety of factors. In the coastal areas of China, groundwater has gradually become salinized into brine, which inevitably alters the original microstructure of rocks and affects the stability of underground structures. To clarify the evolution of the rock microstructure under brine erosion, this study used NMR technology to investigate the pore evolution characteristics of red sandstone under brine erosion. The experimental results show that the water absorption capacity of sandstone is influenced by the solution environment, with the lowest absorption rate occurring in regard to brine. The pores in red sandstone undergo significant changes after brine erosion. Factors such as the composition of the brine and soaking time affect sandstone porosity, with transformations of mini-pores and meso-pores leading to changes in porosity. In addition, XRD tests were carried out on the soaked red sandstone samples to analyze the changes in the main mineral components of the sandstone after brine erosion.

## 1. Introduction

As urbanization progresses, groundwater in coastal areas of China gradually salinizes into brine. Compared with ordinary groundwater, brine has a mineralization level reaching TDS ≥ 1.0 g/L. This brine not only affects urban water supplies, but also impacts the stability of underground structures and even the safety of mineral extraction in coastal bedrock. Taking the Sanshandao Gold Mine in Laizhou Bay, China, as an example, detection shows that most of the bedrock water in this mining area is highly mineralized brine [1,2], with a TDS of up to 92.75 g/L [3]. Rock is a typical heterogeneous material, with complex internal microstructures, such as microfractures, pores, mineral interfaces, and structural cracks. These internal defects not only cause significant anisotropy and heterogeneity in regard to the physical and mechanical properties of rock, but also make it prone to interactions with external media. Brine in bedrock is primarily stored within the fractures and pores of the rock, where it inevitably undergoes physical and chemical interactions [4], altering the original microstructure of the rock. This leads to changes in the rock’s properties that adversely affect underground structure stability [5,6,7]. Therefore, studying the evolution of the microstructure of bedrock under brine erosion is of great practical significance for enhancing the safety of underground structures.

Current studies on rock microstructure often indirectly reflect changes in the rock’s microstructure by measuring variations in certain microparameters of the rock. Among these, porosity is an important parameter for characterizing the rock’s microstructure [8]. Because porosity is strongly correlated with rock strength [9,10], brittleness [11], and other properties, measuring rock porosity can link the microstructure to macroscopic properties. Presently, methods for measuring rock porosity include the use of a mercury intrusion porosimeter [12], the gas adsorption method (BET) [13,14], CT scanning [15], and nuclear magnetic resonance (NMR) [16,17]. As a nondestructive testing method, NMR technology is often used to measure the porosity of rock materials under various conditions [18]. For example, Zhang et al. [18] used NMR technology to study the evolution of rock microstructure in a long-term acidic environment; Yu et al. [19] investigated the damage evolution of the coal microstructure under different dry–wet cycles; Li et al. [20] used NMR technology to study the pore structure of sandstone after high-temperature treatment; Gong et al. [21] employed NMR technology to research pore structure evolution and frost damage in granite during cyclic freeze–thaw processes. Meanwhile, many scholars have also investigated the effect of the type of iron-containing ion minerals and the form of their assignment on the transverse relaxation time, T_2_, of rocks [22,23]. Washburn et al. [24] proposed a method for the fast and quantitative prediction of the relaxation rate of sandstone surfaces, using laser-induced spectroscopy. They found that paramagnetic impurities, such as iron and manganese, have a significant effect on the relaxation rate.

Extensive research on the effects of water on rocks has shown that the water content [25] and dry–wet cycles [26] both affect rock porosity. Moreover, numerous findings indicate that chemical solutions also influence rock porosity. For instance, the study by Katika et al. [27] showed that saturated solutions containing divalent ions affect the porosity of limestone. Yu et al. [28] discovered that under the same soaking time, a lower solution pH leads to higher limestone porosity, a result that was similarly found by Li et al. [29]. Li et al. [30] examined changes in rock porosity after chemical erosion and concluded that porosity changes are the fundamental cause of mechanical property degradation. Feng et al. [31] immersed sandstone specimens in alkaline solutions, comparing the microtopography and mineral composition of sandstone before and after corrosion. Zuo et al. [32] found that during CO_2_-saturated saline immersion, the minerals in rock dissolve and the rock porosity rapidly increases. Cao et al. [33] carried out dry–wet cycle tests on sandstone in a sodium chloride solution, which showed an increase in porosity according to the number of dry–wet cycles. Shao et al. [34] demonstrated that salt dissolution promotes connectivity among shale pores. T. Heggheim et al. [35] prepared artificial saltwater enriched with Ca^2+^ to investigate weakening and damage mechanisms in limestone after immersion in the artificial saltwater, discussing rock weakening from the perspectives of chemical dissolution and precipitation processes, as well as the chemistry of thin water films near intracrystalline structures.

However, there are still some gaps in previous research. While there has been extensive research on the effects of water on rock porosity, much of it focuses on the impact of water itself, such as the water content of rocks, with little consideration given to the influence of other chemical components in the water. In reality, the water encountered in geotechnical engineering is a complex mixture that contains various chemical components, including groundwater with a significant amount of salt minerals. Research on the effects of salt solutions on rock porosity is relatively scarce and, of those studies that do, they often only examine the effects of single chemical components, such as sodium chloride, which clearly does not reflect actual field conditions. Based on the above research gaps, this study prepared brine in a laboratory and separately prepared three groups of single-component salt solutions, according to the components of the brine. Red sandstone specimens were immersed in these solutions for 14, 21, and 35 days. After immersion, NMR and XRD tests were conducted on the red sandstone to assess the evolution of its microstructure and the variation in the content of its main mineral components.

## 2. Experimental Materials and Preparation

### 2.1. Specimen Preparation

**Figure 1:**
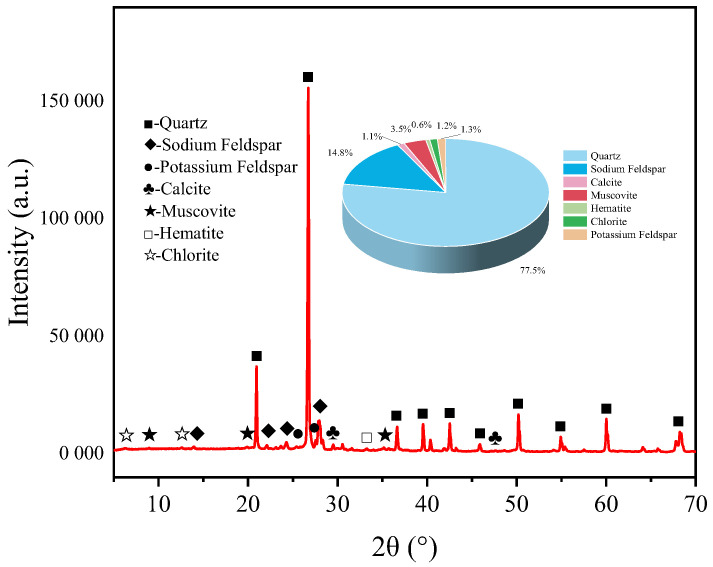
XRD pattern.

Red sandstone, as a common rock material, is widely utilized in studies of rock properties [36]. In this research, red sandstone was selected as the test material. Basic mechanical tests and polarized light examinations were performed on the chosen specimens. The selected red sandstone has a density of 2.35 g/cm^3^ and an average P-wave velocity of 2728 m/s. Its strength properties are as follows: a uniaxial compressive strength of 74.52 MPa, a tensile strength of 4.63 MPa, an elastic modulus of 13.47 GPa, and a Poisson’s ratio of 0.28. According to the XRD test results, the main mineral components in the selected red sandstone include quartz, albite, calcite, hematite, muscovite, chlorite, and orthoclase. The specific contents of these minerals are shown in Figure 1.

### 2.2. Preparation and Soaking Scheme of Brine Solution

In the laboratory, brine was prepared using deionized water instead of tap water to avoid ion interference. According to previous research, the typical components of brine include Na^+^, Cl^−^, SO_4_^2−^, Mg^2+^, Ca^2+^, and other ions, with the selected chemical reagents being NaCl, MgSO_4_, and CaCl_2_. Water solutions of these three salts were also prepared separately, for comparison purposes. The red sandstone specimens were immersed for 14, 21, and 35 days. The specific brine solution compositions are shown in Table 1.

### 2.3. NMR Testing and Pore Structure Classification Models

The transverse relaxation time (*T*_2_) is one of the most common measurements in NMR testing; it concerns the decay of the amplitude of the spin-echo of a single pore body [37,38]. The complexity of the pore structure can be evaluated using fractal theory, primarily through the use of the fractal dimension [39,40].

The testing equipment includes the use of the MacroMR12-150H-I low-field nuclear magnetic resonance analysis system from Chongqing University, which is manufactured by Suzhou Niumag Analytical Instrument Corporation, Suzhou, China. The nuclear magnetic resonance test involves the collection of sample signals based on the Carr–Purcell–Meiboom–Gill (CPMG) sequence, with an echo time interval TE of 0.1 ms, a repeat sampling interval Tw of 1.5 ms, 16 scan repetitions Ns, and 10,000 echoes NECH. In regard to the test, the specimens are monolithic and are dried in a drying oven for more than 48 h until there is no change in the quality of the rock specimen. During the tests, the specimens were placed in a dry container until the temperature was reduced to room temperature and the samples were immersed in four solutions. In regard to the NMR test, the specimen was placed horizontally in the middle of the receiver coil at the beginning of the test and the measurement parameters were kept consistent.

### 2.4. Rock Water Absorption Test

In this study, the saturation process of red sandstone was recorded in four different aqueous solutions, namely a NaCl solution, MgSO_4_, a CaCl_2_ solution, and a brine solution. By measuring changes in the mass of the soaked specimens, the water content at different soaking times was calculated. When the water content no longer increases, saturation is reached. The calculation method for the water content is as follows:

(1) $$W_{i}=\frac{\left(M_{i}-{M\;}_{o}\right)}{M_{o}}\times 100\%$$

where $M_{i}$ is the mass of the specimen after soaking, and $M_{o}$ is the mass of the specimen in its natural state.

**Figure 2:**
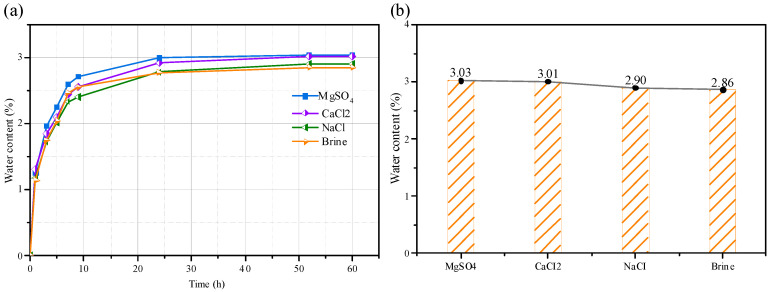
Water absorption characteristics of sandstone in different solutions: (**a**) Water absorption saturation Curve; (**b**) Water content of different solutions.

The measured results are shown in Figure 2. The saturation curves of the sandstone in different aqueous solutions do not exhibit significant differences. The water content curves of the sandstone in different solutions can all be divided into three stages: a rapid increase stage, a stable growth stage, and a constant stage. However, the saturation rate of sandstone in different aqueous solutions shows certain differences. There are also some differences among the various solutions, with the brine group and NaCl group being relatively close in terms of their results, while the MgSO_4_ group and CaCl_2_ group have noticeably lower values.

## 3. Test Results and Analysis

### 3.1. T_2_ Distribution Curve Analysis

**Figure 3:**
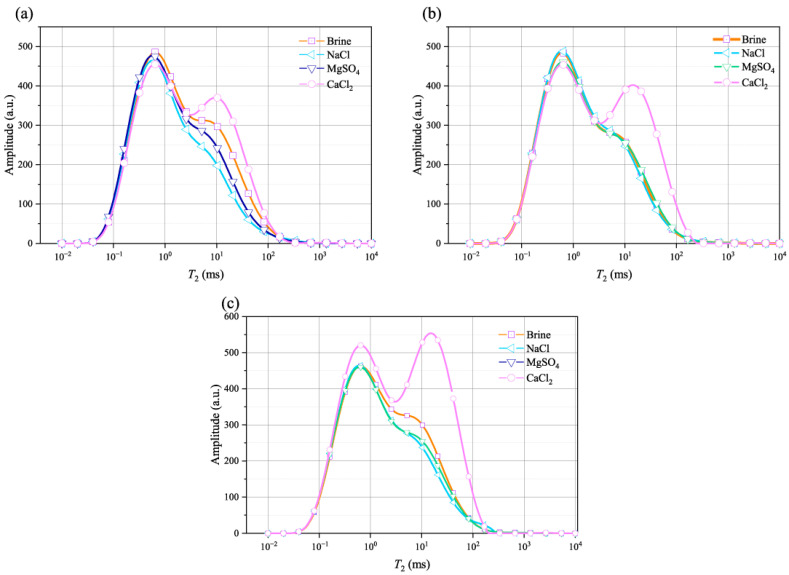
*T*_2_ curves of the rocks after being soaked in different solutions for: (**a**) 14 d; (**b**) 21 d; and (**c**) 34 d.

Previous studies have shown that the *T*_2_ curve obtained through NMR testing can reflect the pore characteristics within rock, indicating the size and number of pores, as well as the connectivity between pores. Figure 3 shows the *T*_2_ curves of the rocks after being soaked in different aqueous solutions for various durations. From the figure, it can be observed that the *T*_2_ curves differ noticeably depending on the type of solution; most curves have only a single peak. However, under the same soaking time, the sample immersed in the NaCl solution exhibits a second peak, indicating that Na^+^ has a strong impact on the rock’s pores, resulting in a higher degree of internal pore connectivity than the other solutions. The aqueous solutions containing Ca^2+^ and Mg^2+^ have essentially the same effect on the sandstone pores, with very similar *T*_2_ curve distributions.

**Figure 4:**
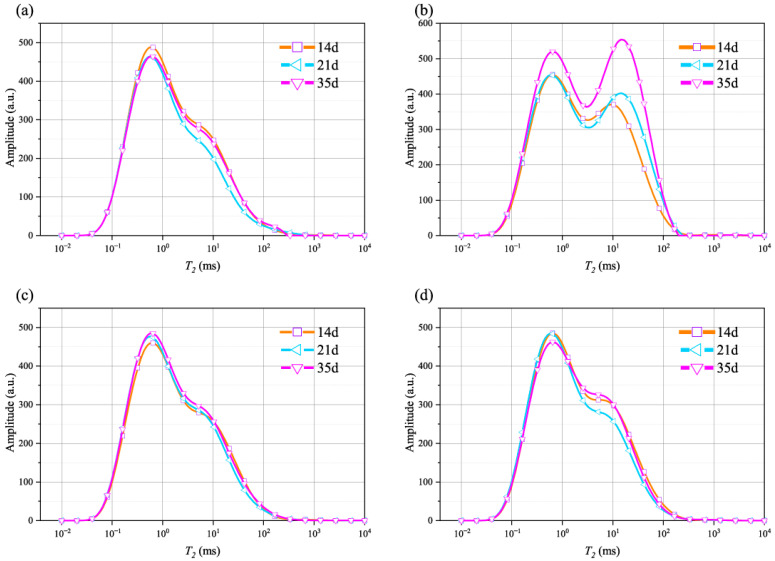
*T*_2_ curves of sandstone after different soaking times in: (**a**) brine; (**b**) NaCl; (**c**) MgSO_4_; and (**d**) CaCl_2_.

In order to better illustrate the effect of the soaking time on the *T*_2_ spectrum curves, Figure 4 presents the *T*_2_ spectrum curves for different soaking times in the same solution. After immersion in different solutions, the impact of time on the *T*_2_ spectrum curve differs. For the rocks immersed in the NaCl solution, as the time increases, the peak of the *T*_2_ spectrum curve rises, and the curve shifts to the right. For the rock samples immersed in the CaCl_2_ solution, the MgSO_4_ solution, and brine, the pattern of the *T*_2_ spectrum curve changes at 21 d, with the peak of the *T*_2_ spectrum curve first decreasing and then increasing.

According to previous research, the pores inside rock can be classified into two types, namely absorbent pores and permeable pores, with the classification standard being the *T*_2_ cutoff value (*T*_2C_) [41]. The water in absorbent pores is referred to as bound water, which remains relatively still, while the water in permeable pores is called free water, which is more mobile. Based on the characteristics of free water, centrifugation can be used to remove the free water from a saturated sample, leaving only the bound water. Additionally, NMR testing of the saturated sample provides the *T*_2_ spectrum of all the pore water, while NMR testing of the centrifuged sample captures the *T*_2_ spectrum of the bound water. In this experiment, the *T*_2_ cutoff value is 10 ms.

The *T*_2_ distribution curve to the left of *T*_2C_ represents the water in the adsorptive pores (bound water), while the right side represents the water in the permeable pores (free water). The area between the *T*_2_ distribution curve and the *x*-axis is referred to as the curve area, which characterizes the pore content.

**Figure 5:**
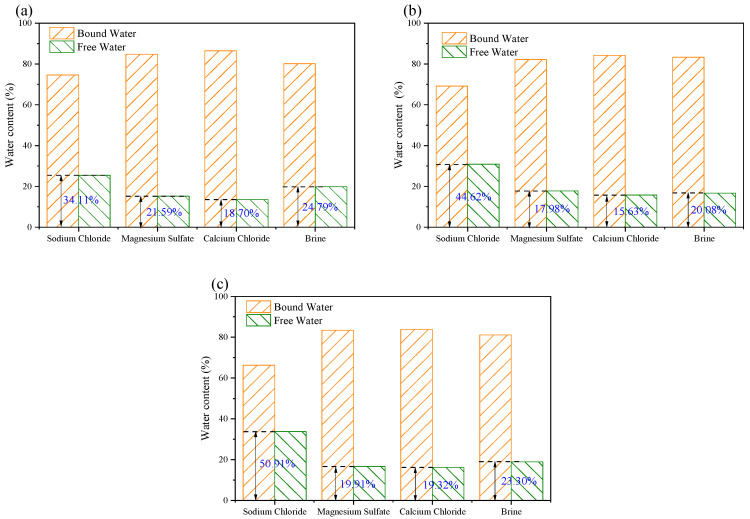
The ratio of free water to saturated water in sandstone after being soaked in different solutions for: (**a**) 14 d; (**b**) 21 d; and (**c**) 35 d.

Figure 5 shows the changes in free water and bound water in the rock after immersion in different solutions. Under the same soaking time, different solutions affect the proportion of free water differently. After immersion in the NaCl solution and brine, the proportion of free water relative to bound water is higher than in the other two solutions, while the proportion of free water after immersion in the MgSO_4_ and CaCl_2_ solutions is relatively close. The soaking time also influences the ratio of free water. As the soaking time in the NaCl solution increases, the pores in the red sandstone continue to expand, and the proportion of free water steadily grows, rising from 34.11% to 50.91%. However, in the samples immersed in MgSO_4_, CaCl_2_, and brine, the lowest proportion of free water occurs at 21 days, with the proportion of free water initially increasing and then decreasing as the soaking time increases. This suggests that ions in the solution react with mineral components in the rock, forming certain minerals that clog the existing pores and, thus, reduce the overall pore space in the rock.

### 3.2. Pore Volume Fraction Distribution

As already mentioned in the introduction, a larger *T*_2_ value corresponds to larger pores in the rock. Previous research classifies pores into micropores, meso-pores, and macro-pores, based on the pore diameter, specifically *r* ≤ 0.1 μm for mini-pores, 0.1 < *r* < 1 μm for meso-pores, and *r* ≥ 1.0 μm for macro-pores [21].

**Figure 6:**
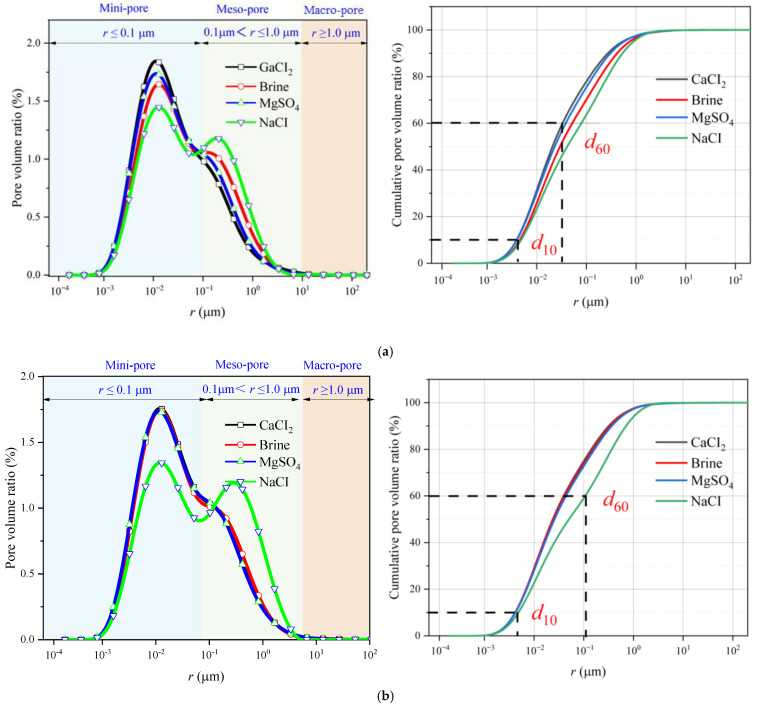

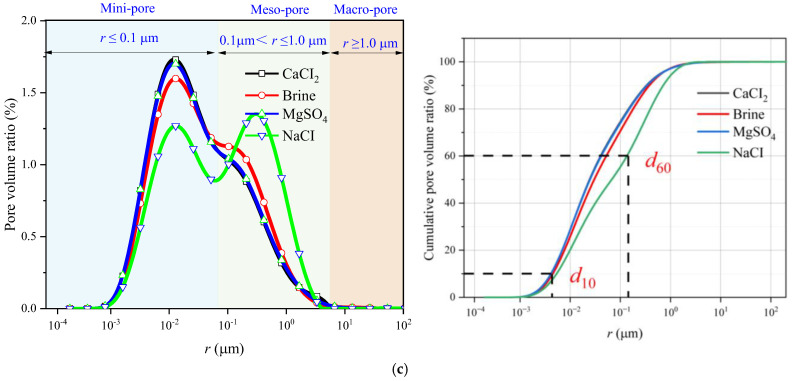
Effect of brine solution on the distribution characteristics of the rock pore volume fraction after immersion for: (**a**) 14 d; (**b**) 21 d; and (**c**) 35 d.

Figure 6 shows how the pore volume fraction and cumulative pore volume fraction vary according to the immersion of the samples in different solutions for different soaking times. It can be observed that the mini-pores and macro-pores in the rocks immersed in brine and the NaCl solutions are more abundant than those in the rocks immersed in the CaCl_2_ and MgSO_4_ solutions. The changes in the cumulative curve better reveal this characteristic, as the cumulative curve shifts toward the specimen treated with the NaCl solution, indicating that NaCl immersion has a more significant influence on the internal pores of the sandstone.

Introducing the concept of the uniformity coefficient from soil particle studies, we may consider the uniformity coefficient of rock pores to be [42]:

(2) $$C_{U}^{P}=\frac{d_{60}}{d_{10}}$$

where $C_{U}^{P}$ represents the heterogeneity coefficient of pores, $d_{60}$ represents the pore diameter corresponding to 60% of the cumulative pore volume, and $d_{10}$ represents the pore diameter corresponding to 10% of the cumulative pore volume.

When $C_{U}^{P}$ ≥ 5, the rock pore distribution is heterogeneous, otherwise the rock pore distribution is homogeneous.

The calculation results of the uniformity coefficient are shown in the figure. It can be seen that after immersion in different solutions, the non-uniformity coefficients of the rock pores are all greater than 5, indicating that the pore distribution inside the rock is generally non-uniform. Under the same soaking time, the uniformity coefficient of the rock pores after immersion in the NaCl solution is significantly higher than that of the other groups, and the effects of the MgSO_4_ and CaCl_2_ solutions on the rock’s pore uniformity coefficient are essentially the same, which is consistent with the previous analysis.

**Figure 7:**
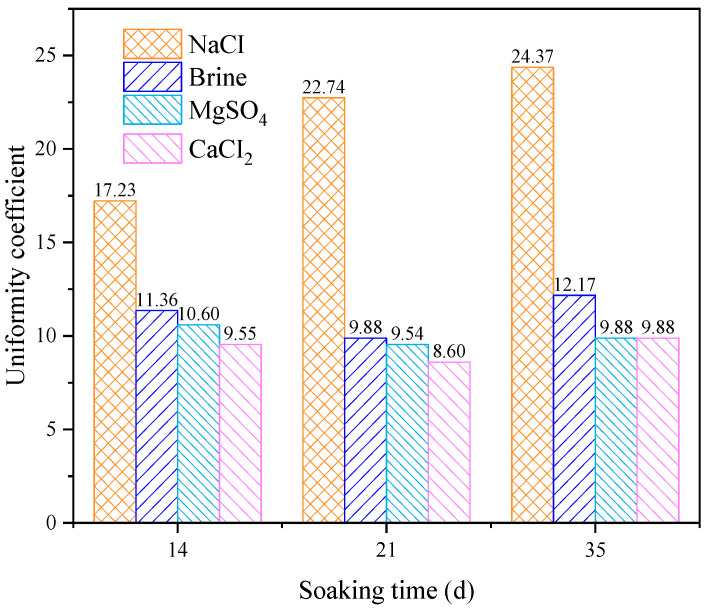
Variation in the characteristics of the pore non-uniformity coefficient in sandstone.

As shown in Figure 7, in regard to the calculated uniformity coefficients of the rock pores, under the same immersion conditions, the pore uniformity coefficient is highest after immersion in the NaCl solution and lowest after immersion in the CaCl_2_ solution. With increasing time, the uniformity coefficient shows an upward trend, but due to the reaction between ions in the solution and mineral components in the rock, certain pore changes occur. To some extent, this inhibits the increase in pore space and plays a suppressive role in regard to water-induced weakening.

Overall, as the time increases, the pore volume ratio curve shifts to the right, indicating that as the soaking time increases, the pore radius within the rock gradually grows. Although different ions react with the mineral components inside the rock, the effect of water on the internal pores of the rock continues to strengthen over time. The calculation results of the uniformity coefficient are shown in the Figure 7. It can be observed that after soaking in different aqueous solutions, the uniformity coefficient of the pores in the rock is greater than 5, indicating that the pore distribution inside the rock is generally uneven. Under the same soaking time, the uniformity coefficient of the pores in the rock after soaking in the sodium chloride solution is significantly higher than that of the other groups. The effects of the magnesium sulfate and calcium chloride solutions on the uniformity coefficient of the pores inside the rock are basically the same, which is consistent with previous analyses.

### 3.3. Porosity

**Figure 8:**
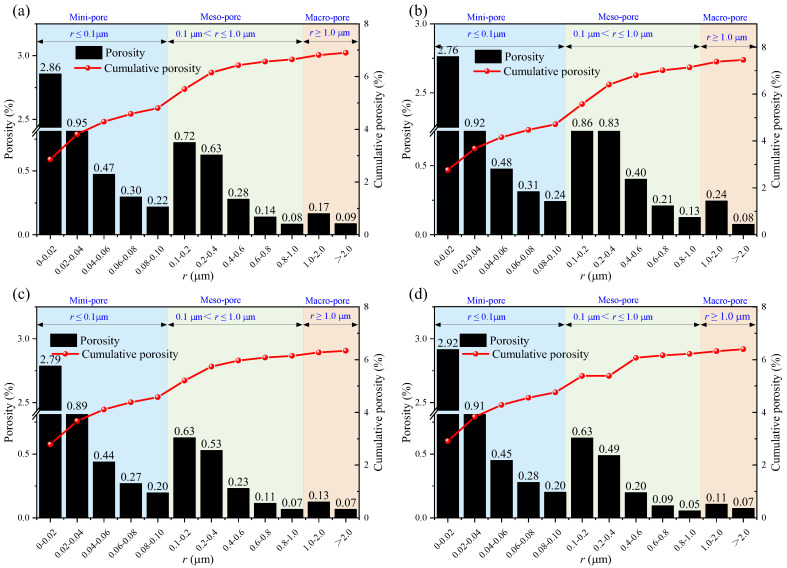
Porosity after 14 days of immersion in: (**a**) brine; (**b**) NaCl; (**c**) MgSO_4_; and (**d**) CaCl_2_.

**Figure 9:**
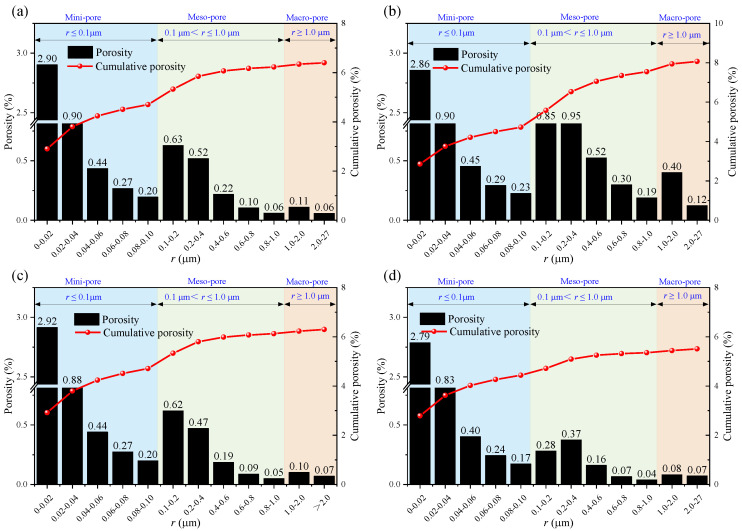
Porosity after 21 days of immersion in: (**a**) brine; (**b**) NaCl; (**c**) MgSO_4_; and (**d**) CaCl_2_.

**Figure 10:**
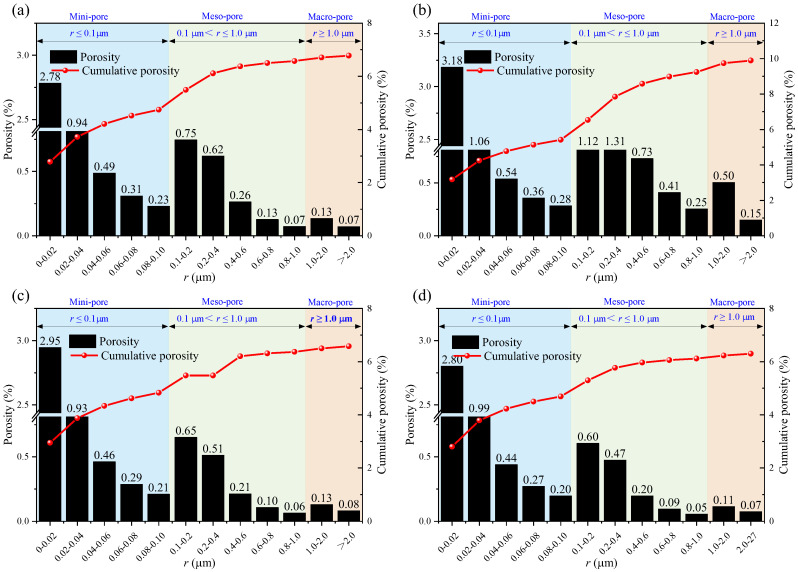
Porosity after 35 days of immersion in: (**a**) brine; (**b**) NaCl; (**c**) MgSO_4_; and (**d**) CaCl_2_.

Nuclear magnetic resonance (NMR) porosity is derived from the cumulative percentage of pores of various diameters. Figure 8, Figure 9 and Figure 10 show the pore distribution for different pore diameters at different soaking times.

**Figure 11:**
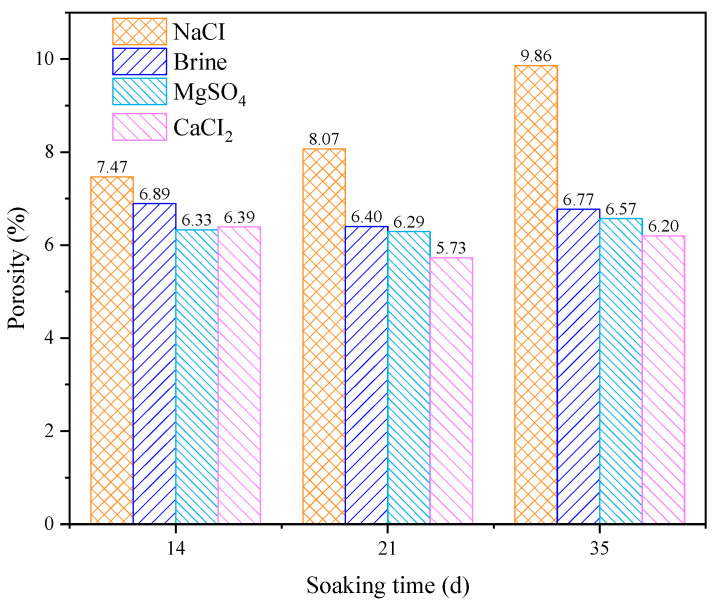
Characteristic changes in sandstone porosity after being soaked in different solutions for various durations.

The porosity results of the sandstone after immersion in brine solution are shown in Figure 11. Under the same immersion duration, compared to brine immersion, red sandstone immersed in the NaCl solution exhibits higher porosity, while immersion in the MgSO_4_ and CaCl_2_ solutions leads to lower porosity. Among these, the specimens immersed in the CaCl_2_ solution show the lowest porosity. The soaking time also has a clear effect on sandstone porosity. After immersion in brine, MgSO_4_, and CaCl_2_ solutions, the rock’s porosity first decreases and then increases over time, indicating the presence of complex mechanisms involving multiple competing factors, including the reaction of solution ions with internal mineral components and the weakening effect of water. However, after immersion in the NaCl solution, the porosity of red sandstone continues to increase over time.

### 3.4. Permeability

Kenyon et al. [43] proposed the Schlumberger-Doll Research (SDR) permeability model, which is a model for measuring sandstone permeability, based on the *T*_2_ spectrum distribution, and is specific to sandstone, as follows:

(3) $$K=C\varphi^{4}T_{2g}^{2}$$

where *K* is the nuclear magnetic resonance permeability, φ is the nuclear magnetic resonance porosity, *C* is the model parameter (usually 4 [44]), and *T*_2g_ is the weighted geometric mean of the *T*_2_ curve.

**Figure 12:**
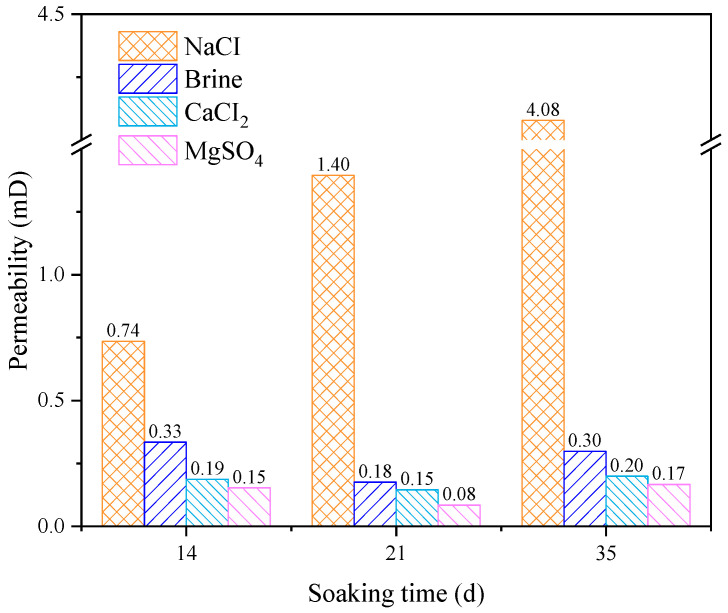
Characteristic changes in sandstone permeability after immersion in different solutions for various durations.

*T*_2g_ is calculated as follows:

(4) $$T_{2g}=T_{1}^{\frac{n_{1}}{n_{1}+n_{2}\cdots +n_{200}}}T_{2}^{\frac{n_{2}}{n_{1}+n_{2}\cdots +n_{200}}}T_{i}^{\frac{n_{i}}{n_{1}+n_{2}\cdots +n_{200}}}\cdots \cdots T_{200}^{\frac{n_{200}}{n_{1}+n_{2}\cdots +n_{200}}}$$

where *i* represents each point in the *T*_2_ spectrum; and *T*_i_ and *n_i_* represent the *T*_2_ relaxation time and the corresponding amplitude of each point, respectively. The calculation results are shown in Figure 12.

The permeability of rocks shows significant differences after being soaked in different aqueous solutions. The permeability of rock immersed in the NaCl solution is significantly higher than that in the other solutions, and this gap continues to widen as the soaking time increases. Overall, as soaking time increases, the rock’s permeability rises. Although the rock’s permeability decreases after 21 days of immersion in the CaCl_2_, MgSO_4_, and brine solutions, it continues to increase with further soaking time.

### 3.5. Tortuosity

The tortuosity of rock pores is a parameter that measures the ratio of the actual flow path of fluids in rock pores to the straight-line distance. It reflects the degree of curvature of the path when fluids pass through the rock pore network. The higher the tortuosity, the more complex the path that the fluid needs to flow along, affecting the rock’s permeability and fluid transport efficiency. Based on the assumption that some particles in certain porous media can overlap indefinitely, while others cannot, Yu et al. [45] established a geometric model for the average tortuosity of porous media, as shown in the following formula:

(5) $$\tau =\frac{1}{2}\left[1+\frac{1}{2}\sqrt{1-\varphi }+\sqrt{1-\varphi }\cdot \frac{\sqrt{{\left(\frac{1}{\sqrt{1-\varphi }}-1\right)}^{2}+\frac{1}{4}}}{1-\sqrt{1-\varphi }}\right]$$

where τ is tortuosity, and φ is NMR porosity.

**Figure 13:**
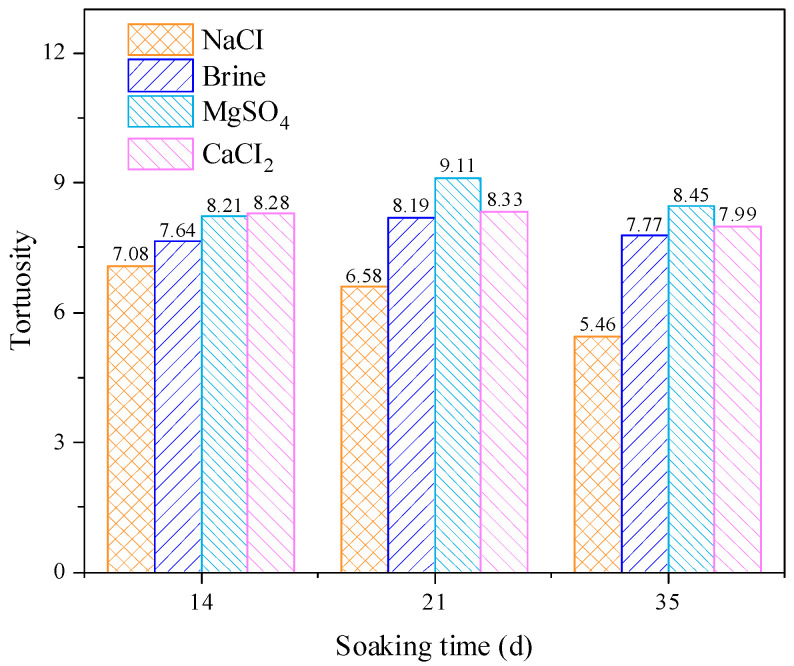
Variation characteristics of sandstone curvature after soaking in different solutions for various durations.

Figure 13 shows the relationship between the tortuosity of the rock specimens and the soaking solution and soaking time. At the same soaking time, the difference in the rock pore tortuosity after soaking in different aqueous solutions is obvious. Among them, the rock pore tortuosity after soaking in the sodium chloride solution is the lowest, and the rock pore tortuosity after soaking in the calcium chloride aqueous solution is the highest. As the soaking time increases, the pore tortuosity of the rock continues to decrease after soaking in the sodium chloride solution, while the pore tortuosity of the other three groups of aqueous solutions first increases and then decreases. In addition, the curve of the calcium chloride aqueous solution is always in the middle of the other three groups, which is also consistent with other parameter changes.

### 3.6. Fractal Dimension of Pore Structure

**Figure 14:**
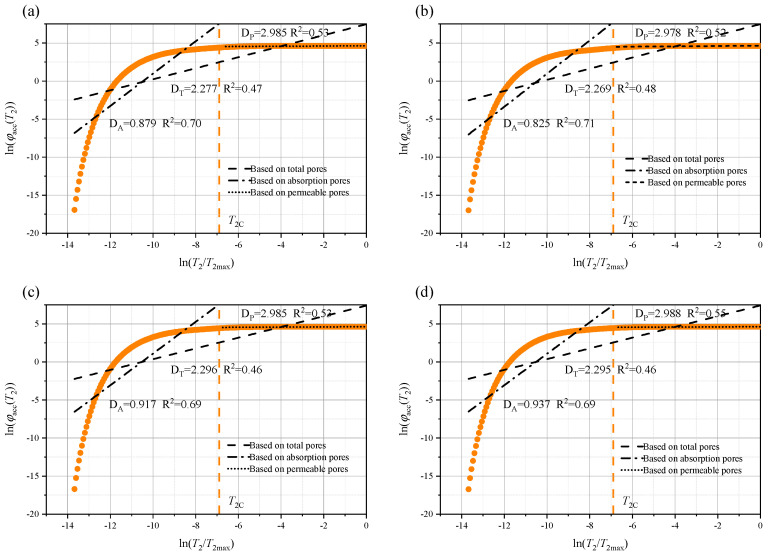
Fractal characteristics of the pore structure after 14 days of soaking in: (**a**) brine; (**b**) NaCl; (**c**) MgSO_4_; and (**d**) CaCl_2_.

**Figure 15:**
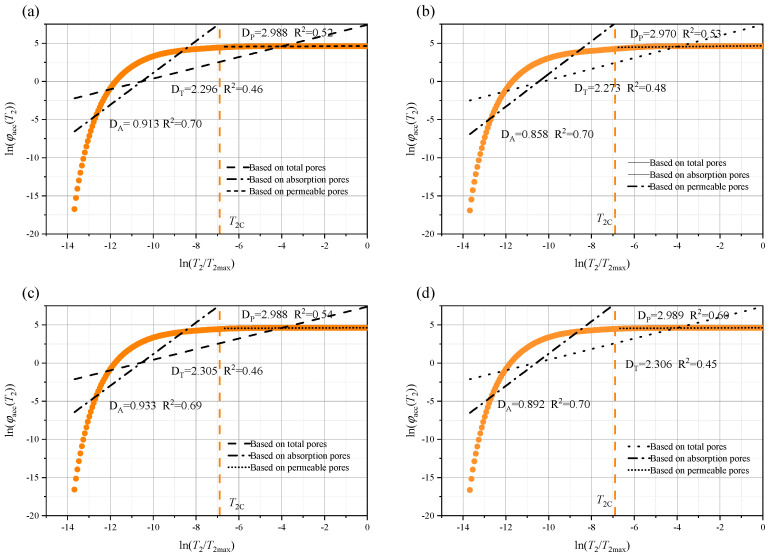
Fractal characteristics of the pore structure after 21 days of soaking in: (**a**) brine; (**b**) NaCl; (**c**) MgSO_4_; and (**d**) CaCl_2_.

**Figure 16:**
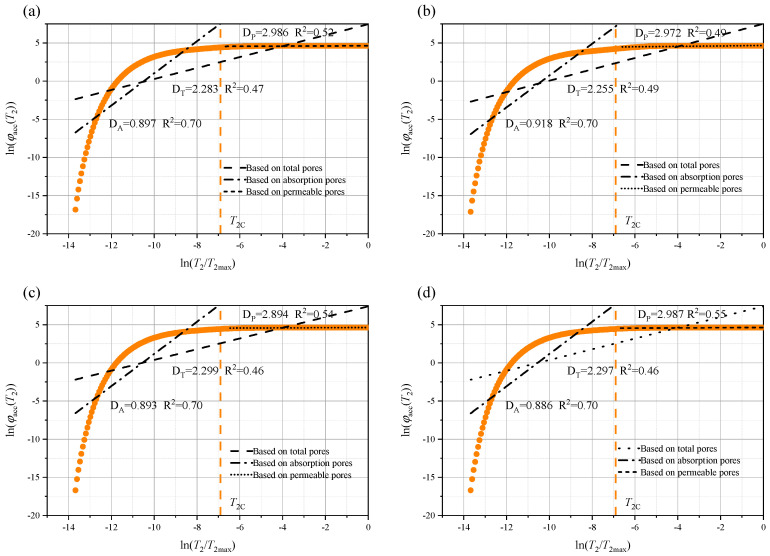
Fractal characteristics of the pore structure after 35 days of soaking in: (**a**) brine; (**b**) NaCl; (**c**) MgSO_4_; and (**d**) CaCl_2_.

Rock, as a typical porous medium, has a pore structure that significantly influences its macroscopic physical and mechanical properties. To further quantify the pore size characteristics of the rock immersed in different aqueous solutions, the fractal dimensions of the total pores (D_T_), permeable pores (D_P_), and adsorbed pores (D_A_) were calculated using Equation (12). In addition, the relationship between ln(φₐcc(*T*_2_)) and ln(*T*_2_/*T*_2_ₘₐₓ) is shown in Figure 14, Figure 15 and Figure 16, where *T*_2_c represents the cutoff value of the transverse relaxation time, set to 10 ms in this study. Notably, D_A_ is much smaller than D_P_. However, from a theoretical standpoint, the three-dimensional pore structure, D, of the rock should range from 2.0 (smooth surface) to 3.0 (highly complex volume) [17,21]. Therefore, D_A_ may not provide a meaningful characterization.

**Figure 17:**
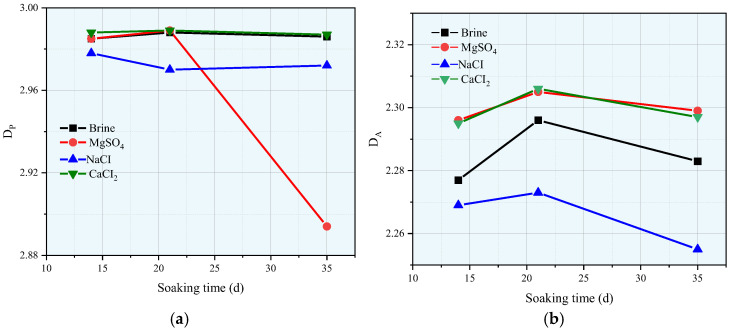
Change characteristics of sandstone pore fractal dimensions after being soaked in different solutions for various durations: (**a**) The law of change of D_P_ with soaking time; (**b**) The law of change of D_A_ with soaking time.

Figure 17 shows the relationship between the fractal dimensions of the permeable pores (D_P_) and adsorbed pores (D_A_) with the soaking time and solution type. From Figure 17a, it can be seen that after the rock is immersed in brine, the magnesium sulfate solution, and the calcium chloride solution, the pore fractal dimension, D_p_, first increases and then decreases with the soaking time. Compared with the immersion in the other solutions, the immersion of the samples in sodium chloride solution also shows that the D_p_ first increases and then decreases with the soaking time. From Figure 17b, it can be seen that after the rock is immersed in all four aqueous solutions, the pore fractal dimension, D_A_, increases first and then decreases as the soaking time grows. Overall, after immersion in sodium chloride, both fractal dimensions of the rock are the lowest, whereas after immersion in brine, both fractal dimensions are at a middle level. Changes in the pore fractal dimension characteristics after immersion in different solutions can be attributed to chemical reactions, in which the solution components and the mineral components inside the rock undergo complex reactions that disrupt mineral particles, crystals, and their connections. These reactions lead to the reorganization of the microstructure and changes in the complexity of the pore structure.

## 4. Discussion

### 4.1. Evolution of the Pore Structure of Sandstone Eroded by Brine

**Figure 18:**
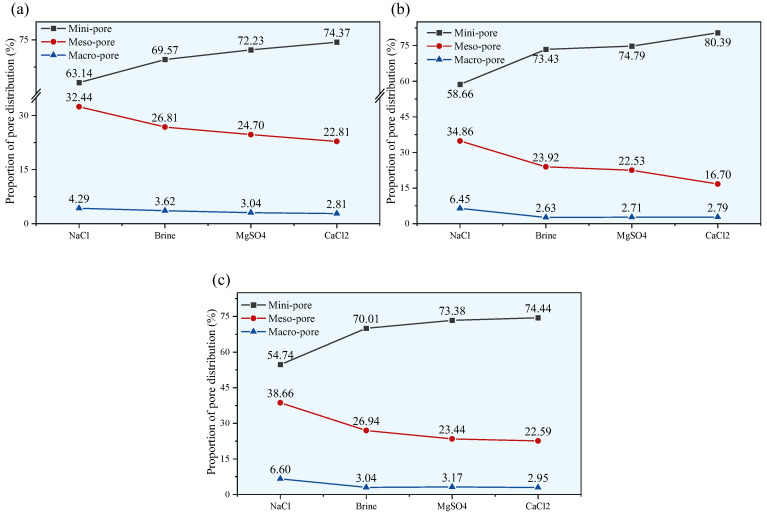
Sandstone pore size distribution for different soaking times: (**a**) 14 d; (**b**) 21 d; and (**c**) 35 d.

To better understand the impact of brine erosion on the microstructure of red sandstone, the evolution pattern of the proportion of pores of different sizes is shown in Figure 18. From the figure, it can be clearly seen that the pore structure of sandstone mainly consists of mini-pores and meso-pores, and the overall proportion of macro-pores is not very high. Therefore, the higher the sandstone’s porosity, the smaller the proportion of small pores, and the greater the proportion of medium and large pores. The rate of change in the proportion of mini-pores and meso-pores is noticeably higher than that of macro-pores, indicating that changes in porosity after brine immersion are mainly caused by alterations to mini-pores and meso-pores.

Under the same soaking time, compared with the brine solution, the NaCl group has a smaller proportion of mini-pores and a significantly higher proportion of meso-pores and macro-pores. The effects caused by the MgSO_4_ and CaCl_2_ solutions are relatively similar. Regarding this result, there may be several reasons for this. First, it should be noted that NaCl makes up the largest percentage of the brine solution, and previous research has shown that the mechanical properties of sandstone soaked in NaCl solutions at different concentrations vary. The concentrations of the other two solutions are not as high as that of the NaCl solution, so their impact on the sandstone’s microstructure is not very pronounced. Of course, further studies on the effects of different solution components on the microstructure of red sandstone require using solutions with the same concentration, which is a task for our future work. This study mainly explores how a brine solution and its main components affect the microstructure of sandstone; the specific impact of individual ions is not the focus here.

For the same solution, as the soaking time increases, overall, the mini-pores in the sandstone tend to transform into meso-pores and macro-pores, thereby increasing the sandstone’s porosity. Naturally, between 14 and 21 days of immersion, due to mineral precipitation, the proportion of small pores in the brine, MgSO_4_, and CaCl_2_ groups increases, but as the soaking time continues to increase, this trend is suppressed.

### 4.2. Changes in the Mineral Component Content of Sandstone

**Figure 19:**
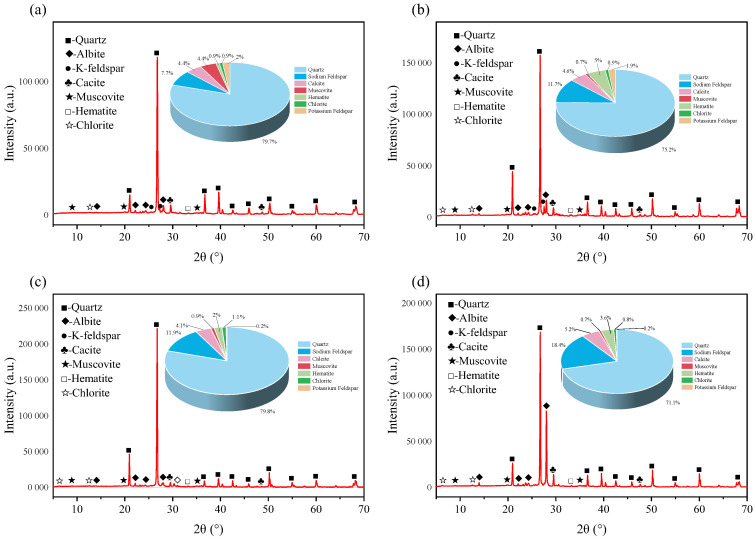
X-ray diffraction patterns of red sandstone soaked for 14 days in: (**a**) brine; (**b**) NaCl; (**c**) MgSO_4_; and (**d**) CaCl_2_.

**Figure 20:**
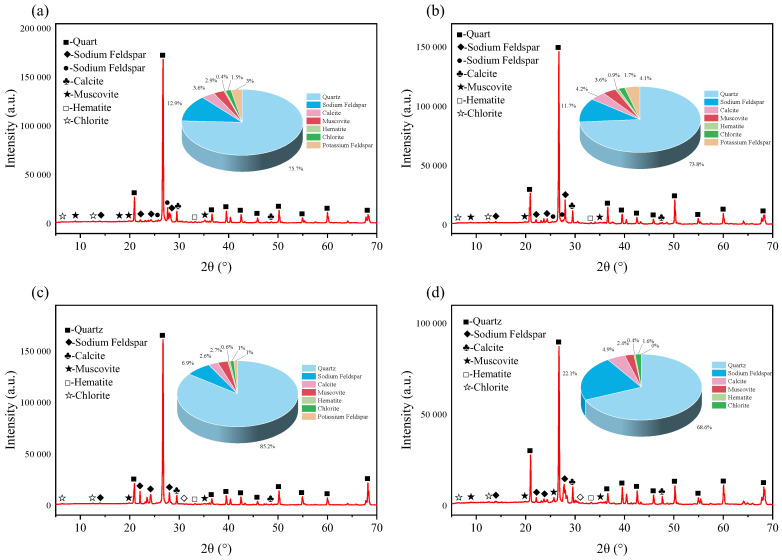
X-ray diffraction patterns of red sandstone soaked for 21 days in: (**a**) brine; (**b**) NaCl; (**c**) MgSO_4_; and (**d**) CaCl_2_.

**Figure 21:**
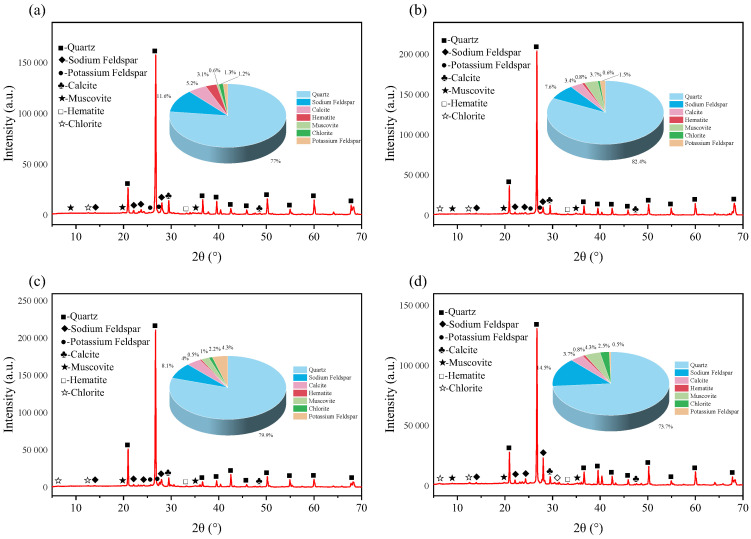
X-ray diffraction patterns of red sandstone soaked for 35 days in: (**a**) brine; (**b**) NaCl; (**c**) MgSO_4_; and (**d**) CaCl_2_.

Figure 19, Figure 20 and Figure 21, respectively, show the XRD diffraction patterns of the internal mineral components of the samples immersed for 14, 21, and 35 days. The XRD diffraction patterns of the specimens after immersion in different aqueous solutions are basically the same, with the main differences lying in the peak values of each mineral in the rock. In regard to an XRD diffraction pattern, the longer and narrower the diffraction peak, the more complete the structure and the higher the degree of crystallinity. The maximum diffraction peak of minerals inside the rock shows certain differences after immersion in different aqueous solutions. Under immersion in the same solution, different soaking times can also alter the rock’s maximum diffraction peak, indicating that both the solution type and the soaking time affect the internal mineral components of the rock. The test results show that the main mineral components of the samples are quartz, sodium feldspar, calcite, and muscovite, collectively accounting for more than 90% of the total composition. Therefore, the subsequent analyses focus on the changes in these four mineral components. Previous studies have shown that the mineral components in rock can react with different aqueous solutions, leading to changes in the rock’s mineral composition. Under the same soaking time, there are variations in the internal mineral components after immersion in each group of solutions; here, the sample immersed for 14 days is used as an example.

After immersion in the four groups of aqueous solutions, the mineral compositions of the specimens showed significant differences. The quartz content in the rock after immersion in brine and magnesium sulfate was the highest, approaching 80%, while immersion in the calcium chloride solution resulted in the lowest quartz content. In the unsoaked specimens, the quartz content was 77.5%. This indicates that complex chemical reactions occurred between the internal mineral components of the rock and the ions in the solution, causing changes in the rock’s original mineral composition. After immersion in the calcium chloride solution, the sodium feldspar content in the specimen reached as high as 18.4%, whereas immersion in brine resulted in a sodium feldspar content of only 7.7%, and the unsoaked specimen had a sodium feldspar content of 14.8%. As for the calcite content, after immersion in the four groups of aqueous solutions, such content was generally in the range of 4–5%, with the calcium chloride group reaching 5.2%, whereas the unsoaked specimen contained 3.3%. Changes in the muscovite content were also relatively complex and varied significantly among the different solutions. After immersion in the magnesium sulfate solution, the muscovite content was as low as 2%, while after immersion in the sodium chloride solution, it reached 5%; in the unsoaked specimen, the percentage of muscovite was 3.5%.

**Figure 22:**
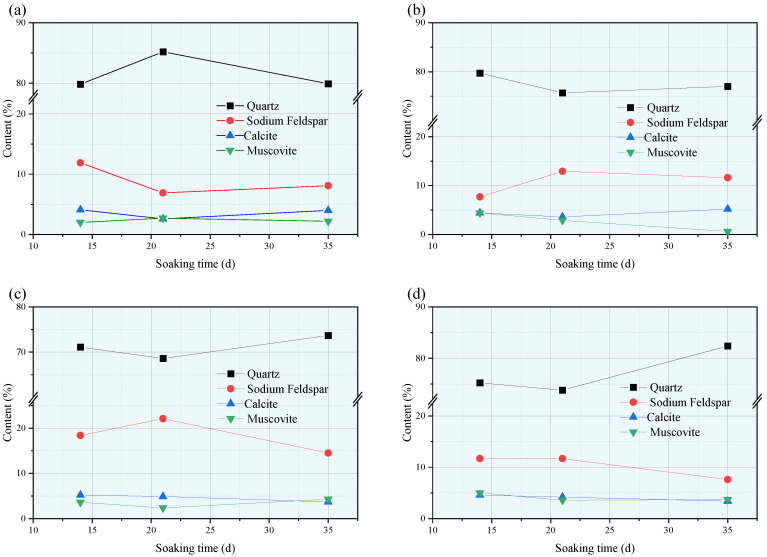
Changes in major mineral components in rock samples for different soaking times in: (**a**) brine; (**b**) NaCl; (**c**) MgSO_4_; and (**d**) CaCl_2_.

The duration of soaking rock specimens in water also affects the changes in their mineral composition. By analyzing the same aqueous solution for different soaking times, the study mainly focuses on the changes in the content of four minerals: quartz, albite, calcite, and muscovite. The results are shown in the Figure 22. Comparing the results of the four groups of aqueous solutions, it can be observed that after soaking in a calcium chloride solution, the quartz content initially increases with time and then decreases. In the other three groups of solutions, the quartz content decreases first and then increases with time. Additionally, the trends in the quartz content and albite content exhibit opposite patterns with an increase in the soaking time, which is consistent across all four groups of specimens: as the quartz content increases, the albite content decreases. The relationships between other major mineral components and the soaking time are generally similar to the trend in the quartz content, mostly decreasing first and then increasing with prolonged soaking time.

After soaking in a calcium chloride solution, the changes in the quartz content and albite content are particularly significant. It is worth noting that under normal temperature and pressure conditions, and when only the calcium chloride solution is used for soaking, quartz (SiO_2_) itself is difficult to dissolve or transform on a large scale. Therefore, the “decrease in quartz content” often does not truly indicate the actual reduction or disappearance of quartz. Instead, it is more likely caused by the following scenarios: a relative decrease in the intensity of the quartz diffraction peaks in the XRD analysis, which then reflects a “decrease in quartz content” in the quantitative analysis. Although quartz is generally stable, if there are microcracks, defect zones, or areas of concentrated mechanical stress on the surface of the rock, extremely slight local dissolution or peeling may occur after long-term soaking [46,47].

## 5. Conclusions

This study focuses on red sandstone. Brine solutions were prepared in the laboratory, and three groups of sandstone specimens subjected to different soaking times were produced. The water absorption properties of the rock were measured. NMR testing and XRD component analysis were then performed on the immersed sandstone specimens to examine the effects of different brine solutions on the microstructure of red sandstone. The main conclusions are as follows:

(1) The water absorption performance of sandstone in aqueous solutions is influenced by the solution’s components; the more complex the composition of the solution, the lower the sandstone’s water absorption performance.
(2) After being soaked in brine, the sandstone’s porosity first increases and then decreases with the soaking time, with the minimum porosity occurring at 21 days. Changes in rock porosity are mainly due to the transformation of mini-pores and meso-pores, whereas the proportion of large pores remains relatively stable. The mineral content of sandstone also changes with an increase in the soaking time.
(3) Different brine components affect the pore and mineral content of sandstone. The pattern of pore changes after immersion in the NaCl solution differs from that of brine, while the patterns observed with the MgSO_4_ and CaCl_2_ solutions are the same as those seen with brine.

## Figures and Tables

**Table 1:** Solution composition and soaking scheme.

Solution Type	Content of Each L Solution Components			Soaking Time
	NaCl (mol/L)	MgSO_4_ (mol/L)	CaCl_2_ (mol/L)	
Brine	2.0	0.3	0.2	14, 21, 35 d
NaCl	2.0	0	0	14, 21, 35 d
MgSO_4_	0	0.3	0	14, 21, 35 d
CaCl_2_	0	0	0.2	14, 21, 35 d

## Data Availability

The original contributions presented in this study are included in the article. Further inquiries can be directed to the corresponding authors.

## References

[1] Yan B.Q., Ren F.H., Cai M.F., Qiao C. (2020). Bayesian Model Based on Markov Chain Monte Carlo for Identifying Mine Water Sources in Submarine Gold Mining. J. Clean. Prod..

[2] Chen B., Yu Y., Su Q., Yang L., Fu T., Liu W., Chen G., Lyu W. (2023). The Study on the Genesis of Underground Brine in Laizhou Bay Based on Hydrochemical Data. Water.

[3] Peng K., Li X.B., Wang Z.W. (2015). Hydrochemical Characteristics of Groundwater Movement and Evolution in the Xinli Deposit of the Sanshandao Gold Mine Using FCM and PCA Methods. Environ. Earth Sci..

[4] Tale F., Kalantariasl A., Shabani A., Abbasi S., Zohoorian A.H., Khamehchi E. (2020). Experimental and Simulation Study of Low Salinity Brine Interactions with Carbonate Rocks. J. Pet. Sci. Eng..

[5] Gu H.Y., Ma F.S., Guo J., Li K.P., Lu R. (2018). Assessment of Water Sources and Mixing of Groundwater in a Coastal Mine: The Sanshandao Gold Mine, China. Mine Water Environ..

[6] Peng K., Liu X., Huang W.S., He H.Y., Ren J., Luo S. (2025). The Role of Minimum Principal Stress in Tunnel Strainburst Considering Spatial Structure Effect. Theor. Appl. Fract. Mec..

[7] Peng K., Liu X., Jing M., Wu T., Luo K., Luo S. (2025). Impact-Water Interaction Effects on Mechanical and Energy Characteristics of Granite during Uniaxial Compression. Geoenergy Sci. Eng..

[8] Anovitz L.M., Cole D.R. (2015). Characterization and Analysis of Porosity and Pore Structures. Rev. Mineral. Geochem..

[9] He M.M., Zhang Z.Q., Zhu J.W., Li N. (2022). Correlation Between the Constant Mi of Hoek–Brown Criterion and Porosity of Intact Rock. Rock Mech. Rock Eng..

[10] Xue L., Qi M., Qin S.Q., Li G.L., Li P., Wang M. (2015). A Potential Strain Indicator for Brittle Failure Prediction of Low-Porosity Rock: Part I—Experimental Studies Based on the Uniaxial Compression Test. Rock Mech. Rock Eng..

[11] Heidari M., Khanlari G.R., Torabi-Kaveh M., Kargarian S., Saneie S. (2014). Effect of Porosity on Rock Brittleness. Rock Mech. Rock Eng..

[12] Zhang N., Zhao F.F., Guo P.Y., Li J.B., Gong W.L., Guo Z.B., Sun X.M. (2018). Nanoscale Pore Structure Characterization and Permeability of Mudrocks and Fine-Grained Sandstones in Coal Reservoirs by Scanning Electron Microscopy, Mercury Intrusion Porosimetry, and Low-Field Nuclear Magnetic Resonance. Geofluids.

[13] Zheng S.J., Yao Y.B., Liu D.M., Cai Y.D., Liu Y. (2018). Characterizations of Full-Scale Pore Size Distribution, Porosity and Permeability of Coals: A Novel Methodology by Nuclear Magnetic Resonance and Fractal Analysis Theory. Int. J. Coal Geol..

[14] Medina-Rodriguez B.X., Frouté L., Alvarado V., Kovscek A.R. (2023). Multimodal Study of the Impact of Stimulation pH on Shale Pore Structure, with an Emphasis on Organics Behavior in Alkaline Environments. Fuel.

[15] Wang S., Wang L.Q., Zhang W.G., Lin S.C., Zhang Y.M., Yang Y., Wang P.Q., Chen L. (2025). Fracture Mode of Water-Immersed Sandstone Based on Micro CT and Statistical Damage Model under Triaxial Compression. Eng. Fract. Mech..

[16] Zeng Z., Ma H., Yang C., Zhao K., Wang X., Zheng Z. (2024). Characterizing Imbibition and Void Structure Evolution in Damaged Rock Salt under Humidity Cycling by Low-Field. Eng. Geol..

[17] Zhang H.X., Sun W., Xie Q., Chen Y., Tu Z., Ban Y. (2024). Study on Mechanical Properties and Damage Characteristics of Acid Corrosion in Granite Based on NMR Technology. Eng. Geol..

[18] Doughty D.A., Tomutsa L. (1997). Imaging Pore Structure and Connectivity by High Resolution NMR Microscopy. Int. J. Rock Mech. Min. Sci..

[19] Yu L.Q., Yao Q.G., Chong Z.H., Li Y.H., Xu Q., Xie H.G., Ye P.Y. (2022). Mechanical and Micro-Structural Damage Mechanisms of Coal Samples Treated with Dry–Wet Cycles. Eng. Geol..

[20] Li M., Wang D.M., Shao Z.L. (2020). Experimental Study on Changes of Pore Structure and Mechanical Properties of Sandstone after High-Temperature Treatment Using Nuclear Magnetic Resonance. Eng. Geol..

[21] Gong Y.F., Song J.X., Wu S.Z., Zhang Y.W. (2024). Evolution of Pore Structure and Analysis of Freeze Damage in Granite during Cyclic Freeze-Thaw Using NMR technique. Eng. Geol..

[22] Jácomo M.H., Trindade R.I.F., Lucas-Oliveira E., Bonagamba T.J. (2020). Magnetic Matrix Effects on NMR Relaxation Times in Sandstones: A Case Study in Solimões Basin. J. Appl. Geophys..

[23] Foley I., Farooqui S.A., Kleinberg R.L. (1996). Effect of Paramagnetic Ions on NMR Relaxation of Fluids at Solid Surfaces. J. Magn. Reson. Ser. A.

[24] Washburn K.E., Sandor M., Cheng Y. (2017). Evaluation of Sandstone Surface Relaxivity Using Laser-Induced Breakdown Spectroscopy. J. Magn. Reson..

[25] Geng J.S., Cao L.W. (2020). Failure Analysis of Water-Bearing Sandstone Using Acoustic Emission and Energy Dissipation. Eng. Fract. Mech..

[26] Xie K., Jiang D., Sun Z., Chen J., Zhang W., Jiang X. (2018). NMR, MRI and AE Statistical Study of Damage Due to a Low Number of Wetting–Drying Cycles in Sandstone from the Three Gorges Reservoir Area. Rock Mech. Rock Eng..

[27] Katika K., Addassi M., Alam M.M., Fabricius I.L. (2015). The Effect of Divalent Ions on the Elasticity and Pore Collapse of Chalk Evaluated from Compressional Wave Velocity and Low-Field Nuclear Magnetic Resonance (NMR). J. Pet. Sci. Eng..

[28] Yu L.Y., Zhang Z.Q., Wu J.Y., Liu R.C., Qin H., Fan P.X. (2020). Experimental Study on the Dynamic Fracture Mechanical Properties of Limestone after Chemical Corrosion. Theor. Appl. Fract. Mech..

[29] Li S.G., Huo R.K., Yoshiaki F.J., Ren D.Z., Song Z.P. (2019). Effect of Acid-Temperature-Pressure on the Damage Characteristics of Sandstone. Int. J. Rock Mech. Min. Sci..

[30] Li H., Zhong Z., Liu X., Sheng Y., Yang D. (2018). Micro-Damage Evolution and Macro-Mechanical Property Degradation of Limestone Due to Chemical Effects. Int. J. Rock Mech. Min. Sci..

[31] Feng X.-T., Chen S., Li S. (2001). Effects of Water Chemistry on Microcracking and Compressive Strength of Granite. Int. J. Rock Mech. Min..

[32] Zou Y.S., Li S.H., Ma X.F., Zhang S.C., Li N., Chen M. (2018). Effects of CO_2_–Brine–Rock Interaction on Porosity/Permeability and Mechanical Properties during Supercritical-CO_2_ Fracturing in Shale Reservoirs. J. Nat. Gas Sci. Eng..

[33] Cao Y., Sun Q., Yang X., Dang C., Geng J. (2022). Sandstone Weathering under Dry–Wet Cycling in NaCl Solution. Bull. Eng. Geol. Environ..

[34] Shao J., You L., Kang Y., Zhang K. (2024). Salt Dissolution and Pore Structure Changes Induced by Fracturing Fluid in Shale Reservoirs. Energy Fuels.

[35] Heggheim T., Madland M.V., Risnes R., Austad T. (2005). A Chemical Induced Enhanced Weakening of Chalk by Seawater. J. Pet. Sci. Eng..

[36] Peng K., Luo K., Wang Y.M., Luo S., Ma T.X., Jing M., Zhang J. (2025). Stress Wave Propagation and Energy Characteristics of Impact-Damaged and Water-Soaked Sandstone with Different Length-to-Diameter Ratios. J. Rock Mech. Geotech..

[37] Bird N.R.A., Preston A.R., Randall E.W., Whalley W.R., Whitmore A.P. (2005). Measurement of the Size Distribution of Water-Filled Pores at Different Matric Potentials by Stray Field Nuclear Magnetic Resonance. Eur. J. Soil Sci..

[38] Talabi O., Blunt M.J. (2010). Pore-Scale Network Simulation of NMR Response in Two-Phase Flow. J. Pet. Sci. Eng..

[39] Pfeifer P., Avnir D. (1983). Chemistry in Noninteger Dimensions between Two and Three. I. Fractal Theory of Heterogeneous Surfaces. J. Chem. Phys..

[40] Paz Ferreiro J., Vidal Vázquez E. (2010). Multifractal Analysis of Hg Pore Size Distributions in Soils with Contrasting Structural Stability. Geoderma.

[41] Li Q., Li X.B., Yin T.B. (2021). Factors Affecting Pore Structure of Granite under Cyclic Heating and Cooling: A Nuclear Magnetic Resonance Investigation. Geothermics.

[42] Jia H., Ding S., Zi F., Dong Y., Shen Y. (2020). Evolution in Sandstone Pore Structures with Freeze-Thaw Cycling and Interpretation of Damage Mechanisms in Saturated Porous Rocks. CATENA.

[43] Kenyon W.E., Day P.I., Straley C., Willemsen J.F. Compact and Consistent Representation of Rock NMR Data for Permeability Estimation. Proceedings of the 61st Annual Technical Conference and Exhibition of the Society of Petroleum Engineers.

[44] Rezaee R., Saeedi A., Clennell B. (2012). Tight Gas Sands Permeability Estimation from Mercury Injection Capillary Pressure and Nuclear Magnetic Resonance Data. J. Pet. Sci. Eng..

[45] Yu B.M., Li J.H. (2004). A Geometry Model for Tortuosity of Flow Path in Porous Media. Chin. Phys. Lett..

[46] Michalske T.A., Freiman S.W. (1982). A Molecular Interpretation of Stress Corrosion in Silica. Nature.

[47] Hadizadeh J., Law R.D. (1991). Water-Weakening of Sandstone and Quartzite Deformed at Various Stress and Strain Rates. Int. J. Rock Mech. Min. Sci. Geomech. Abstr..

